# *Vermamoeba vermiformis* CDC-19 draft genome sequence reveals considerable gene trafficking including with candidate phyla radiation and giant viruses

**DOI:** 10.1038/s41598-020-62836-9

**Published:** 2020-04-03

**Authors:** Nisrine Chelkha, Issam Hasni, Amina Cherif Louazani, Anthony Levasseur, Bernard La Scola, Philippe Colson

**Affiliations:** 1 Aix-Marseille Université, Institut de Recherche pour le Développement (IRD), Assistance Publique - Hôpitaux de Marseille (AP-HM); Microbes, Evolution, Phylogeny and Infection (MEPHI); Institut Hospitalo-Universitaire (IHU) Méditerranée Infection, 27 boulevard Jean Moulin, 13005 Marseille, France; 2 Institut Hospitalo-Universitaire (IHU) Méditerranée Infection, 19-21 boulevard Jean Moulin, 13005 Marseille, France; 3 Amoéba, 38 avenue des Frères Montgolfier, 69680 Chassieu, France

**Keywords:** Environmental microbiology, Clinical microbiology

## Abstract

*Vermamoeba vermiformis* is a predominant free-living amoeba in human environments and amongst the most common amoebae that can cause severe infections in humans. It is a niche for numerous amoeba-resisting microorganisms such as bacteria and giant viruses. Differences in the susceptibility to these giant viruses have been observed. *V. vermiformis* and amoeba-resisting microorganisms share a sympatric lifestyle that can promote exchanges of genetic material. This work analyzed the first draft genome sequence of a *V. vermiformis* strain (CDC-19) through comparative genomic, transcriptomic and phylogenetic analyses. The genome of *V. vermiformis* is 59.5 megabase pairs in size, and 22,483 genes were predicted. A high proportion (10% (n = 2,295)) of putative genes encoded proteins showed the highest sequence homology with a bacterial sequence. The expression of these genes was demonstrated for some bacterial homologous genes. In addition, for 30 genes, we detected best BLAST hits with members of the Candidate Phyla Radiation. Moreover, 185 genes (0.8%) best matched with giant viruses, mostly those related to the subfamily *Klosneuvirinae* (101 genes), in particular Bodo saltans virus (69 genes). Lateral sequence transfers between *V. vermiformis* and amoeba-resisting microorganisms were strengthened by Sanger sequencing, transcriptomic and phylogenetic analyses. This work provides important insights and genetic data for further studies about this amoeba and its interactions with microorganisms.

## Introduction

*Amoebozoa* species are widely distributed in different environments from terrestrial to aquatic ecosystems, where they can play important ecological roles^1,2^. Members of the family *Hartmannellidae* are frequently detected along with a few other amoebae belonging to different genera of the taxon Amoebozoa^3^*. Vermamoeba vermiformis*, a free-living amoeba of the family *Hartmannellidae*, formerly named *Hartmannella vermiformis*, was first isolated in freshwater from the Pigeon Lake, Wisconsin, and the Kankakee River, Indiana (United States)^4^. *V. vermiformis* was thereafter commonly found in fresh surface water^5^, and also in tap water, bottled mineral water, thermal water, and recreational water environments such as fountains and swimming pools^6–8^. Its density in drinking water sources and biofilms is higher than that of *Acanthamoeba castellanii*^9^. *V. vermiformis* has two-stage life, switching between trophozoite and cystic form^3^. Free-living amoebae (FLA) are commonly in contact with animals including humans. *V. vermiformis* was found to be the predominant amoeba in human environments^10,11^, and has been isolated more frequently from different hospital water systems than *Acanthamoeba* spp.^12^. This amoeba is of special interest for human health as it is able, along with other *Amoebozoa* members including *Acanthamoeba* spp., to cause severe infections such as human keratitis^13–15^. Despite its prevalence in human environments and its pathogenicity in humans, the genome of *V. vermiformis* had not been sequenced.

FLA are the niche of several amoeba-resisting microorganisms, including bacteria and fungi. They are potential reservoirs for several human pathogens, including *Salmonella* spp., *Escherichia coli, Shigella* spp., and *Campylobacter* spp., which cause disorders in the human intestinal tract^10,16–18^, and *Legionella pneumophila*, a human pathogen associated with Legionnaires’ disease that can propagate in *V. vermiformis*^19^. Indeed, *V. vermiformis* strain CDC-19 was isolated from a swab sample recovered from a cooling tower in the boiler room of the hospital during a nosocomial legionellosis investigation^20^. Volatile organic compounds have been identified to be involved in the predator-prey interactions between *V. vermiformis* and bacteria, with differences according to the protist-prey partners. Bacterial prey such as *Dyella* sp. and *Collimonas* sp. were recently found to reduce or conversely stimulate the activity of *V. vermiformis*, respectively^21^.

The discovery of the first giant virus, Mimivirus, in the amoeba *Acanthamoeba polyphaga* in 2003, unveiled an unexpected giant virus diversity in different environments^22–25^. In addition to the remarkable sizes of the virions, their genomes were also found to be giant with sizes ranging between about 340 kilobase pairs (kbp) for marseilleviruses and 2,500 kbp for pandoraviruses. These viral genomes had broad gene repertoires reaching more than two thousand genes encoding various functions and many ORFans, and the genetic composition of these viruses far exceeds quantitatively and qualitatively that of known viruses, and rivals that of other small microbes^23^. Moreover, giant viruses have a high level of genome mosaicism, which is likely linked to their sympatric lifestyle in amoebae with other microorganisms, including bacteria, fungi, and virophages^26,27^. Indeed, important sequence exchanges have been observed between giant viruses and both species *Acanthamoeba castellanii* and *Acanthamoeba polyphaga*^28–31^. The majority of described giant viruses have been experimentally isolated from *Acanthamoeba* spp. These amoebae demonstrated differences in their susceptibility to giant viruses, as for the case of pithoviruses and pandoraviruses that were only isolated from *A. castellanii*^32^. Thereafter, different cellular cultures of amoebae other than *Acanthamoeba* spp. have been infected by these giant viruses^33,34^. Furthermore, ten additional isolates of a new giant viral lineage named the faustovirus lineage were obtained from *V. vermiformis*, and their genomes were sequenced. Faustoviruses have icosahedral virions with a diameter of 200–240 nm and are distantly related to the mammalian African swine fever virus^35,36^. Other members of giant virus families were also obtained by co-culturing with *V. vermiformis*, such as Kaumoebavirus found in sewage water^37^, and Orpheovirus IHUMI-LCC2 isolated from a rat stool sample^38^. Abrahão *et al*. discovered the first *Mimiviridae* members, called tupanviruses, that infect both *V. vermiformis* and *A. castellanii*^39^.

Here, for the first time we sequenced the genome of a *V. vermiformis* strain (CDC-19) that has been used for the isolation of giant viruses. Lateral gene transfers with bacteria and giant viruses were also explored.

## Results

### Genome structure and characterization of the putative genes of *V. vermiformis* CDC-19

A total of 41,068,870 and 25,445 reads were obtained by the Illumina MiSeq Nextera XT and the Oxford Nanopore MinION sequencing, respectively, then were used to assemble the *V. vermiformis* CDC-19 genome. Additionally, 1,584,658 reads were obtained by next-generation RNA sequencing on the Illumina MiSeq instrument. A total of 17,244 and 15 scaffolds were obtained by assembling the MiSeq and MinION sequencing products, respectively. Genome coverage was 43X. The draft genome sequence of *V. vermiformis* CDC-19 has a size of 59.5 megabase pairs (Mbp). It encompasses 14,852 scaffolds, with a G + C content of 41.7%. The phylogenetic tree based on 18S rRNA shows that *V. vermiformis* CDC-19 is clustered with other *V. vermiformis* strains (Supplementary Fig. S1). A total of 22,483 putative genes were predicted. The proportion of putative genes with a size equal to or greater than 100 amino acids (aa) was estimated to be 90.3% (20,299 genes). Out of all the predicted genes, 67.9% (15,266) were non-ORFan genes and 32.1% (7,217) were ORFans (i.e. they have no homologs in the NCBI GenBank protein sequence database (nr)) (Table 1). A total of 12,593 genes (56%) were assigned to COG categories (Supplementary Fig. S2). The main functional categories represented were those corresponding to unknown functions (category S (2,829 genes)); signal transduction mechanisms (category T (1,680 genes)); post-translational modifications, protein turnover, chaperones (category O (1,208 genes)); intracellular trafficking, secretion, and vesicular transport (category U (622 genes)); and defense mechanisms (category V (154 genes)) (Supplementary Fig. S2). *V. vermiformis* putative genes have an average of 3.5 introns per gene. This is less than for *A. castellanii* Neff (6.2 introns per gene)^29^. In contrast, the genes putatively derived from lateral sequence transfers have a lower intron composition. On average, the genes best matching with bacteria and archaea have 2.7 introns per gene, and those best matching with giant viruses have 1.4 intron per gene.

**Table 1:** Genomic composition and gene repertoire of *Vermamoeba vermiformis* CDC-19.

Feature	*Vermamoeba vermiformis* CDC-19
Genome size (bp)	59,550,895
GC content (%)	41.7
DNA scaffolds	14,852
Maximum scaffold length (bp)	432,427
Minimum scaffold length (bp)	500
N50 (bp)	7,608
Total number of genes	22,483
Proportion of genes with a size ≥300 bp	20,299
Non-ORFan genes	15,263
ORFan genes	7,220
Genes assigned to COGs	12,593

COG, clusters of orthologous groups of proteins; N50, sequence length of the shortest contig at 50% of the total genome length.

### Taxonomical assignments of *V. vermiformis* CDC-19 genes and identification and analysis of gene trafficking between *V. vermiformis* CDC-19 and bacteria

**Figure 1:**
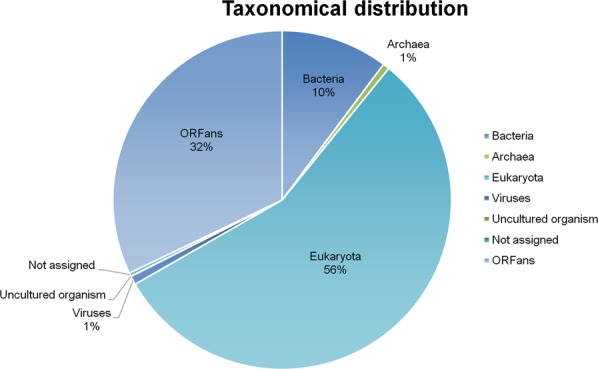
Taxonomical distribution of the *V. vermiformis* CDC-19 predicted proteins.

**Figure 2:**
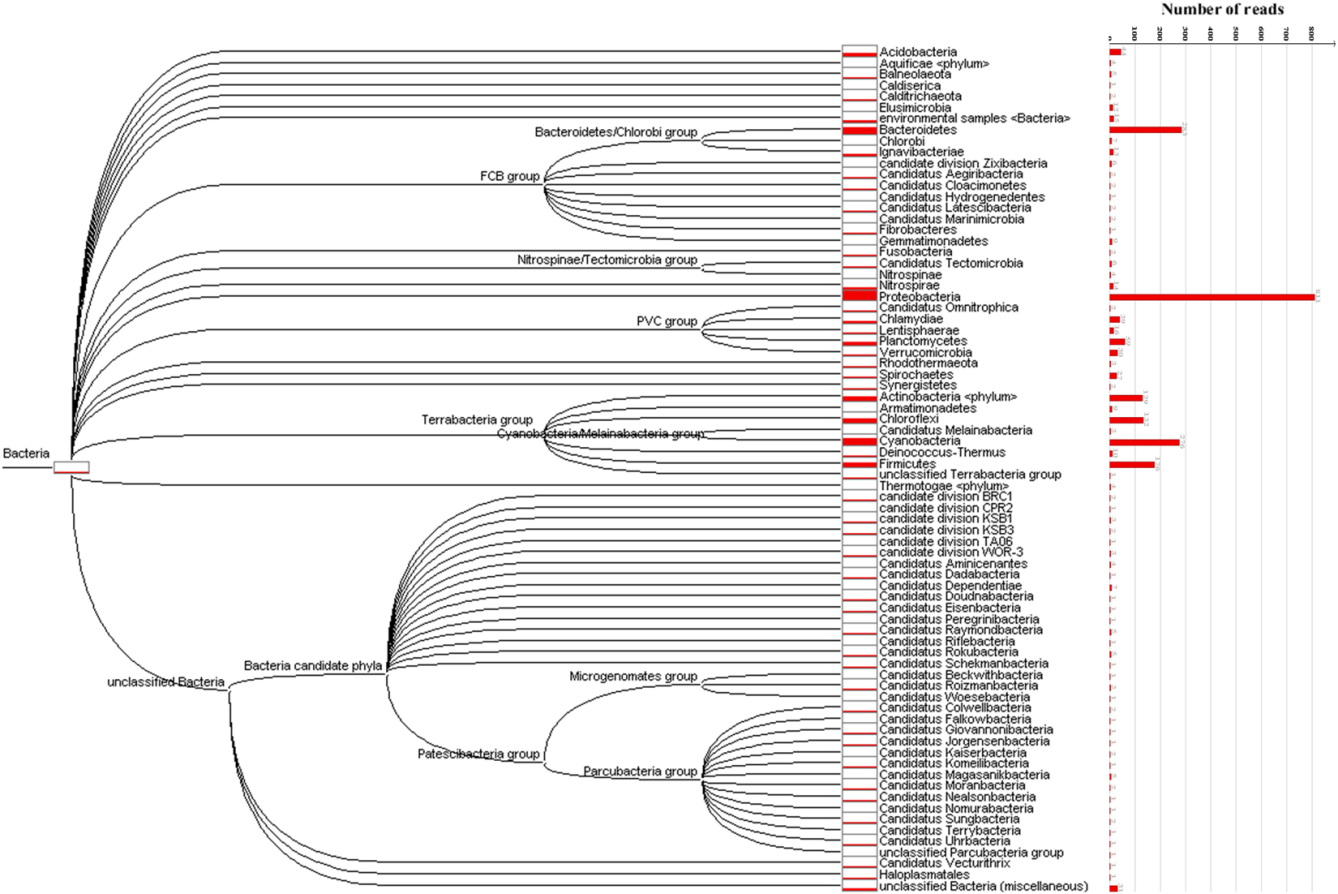
Phylogenetic diversity and number of reads generated from the *V. vermiformis* CDC-19 DNA that best matched with bacteria.

The taxonomical assignment through BLAST searches of genes predicted for *V. vermiformis* CDC-19 showed that 12,567 (55.9%) of them had best hits with eukaryotes, including 4,457 genes best matching with amoebozoan members (19.8%). Also, a high proportion of amoebal genes best matched with bacterial genes (2,295 genes or 10.2%), while 139 (0.6%) and 188 (0.8%) genes had a best hit with archaea and viruses, respectively (Fig. 1). The functional annotation of the *V. vermiformis* CDC-19 putative genes revealed a high proportion of homologous sequences from bacteria, equal to 17.8% of the predicted genes (3,993 genes). Of these 3,993 genes, 2,295 genes were maintained after excluding all suspected contaminant scaffolds, as each of these scaffolds harbored a totality of genes best matching with homologous genes from the same bacteria. For these 2,295 genes, the taxonomical assignment showed that *Proteobacteria* were the most represented with 811 genes (35.3%), followed by *Bacteroidetes* with 283 genes (12.3%), and *Cyanobacteria* with 276 genes (12%) (Fig. 2), compared to 35.4%, 10.5% and 15% for *Acanthamoeba castellanii* strain Neff, respectively^29^. Among these *V. vermiformis* CDC-19 genes best matching with bacteria, 626 (27.3%) were involved in undetermined functions; 164 genes (7.2%) were related to carbohydrate transport and metabolism; 125 genes (5.4%) were related to signal transduction mechanisms; and 97 genes (4.2%) were related to cell wall/membrane/envelope biogenesis (Supplementary Table S1). PCR and Sanger sequencing performed with specific primers designed to target 10 genes among those best matching with bacteria from different phyla were all positive, and one of these genes was found to be expressed and encoded a 1-pyrroline-5-carboxylate dehydrogenase (Supplementary Fig. S3; Supplementary Table S2). Expression of other genes homologous to bacterial genes was detected, such as for the homolog of a tandem-95 repeat protein of *Solitalea canadensis*, which exhibited the highest level of gene expression among genes best matching with bacteria (349 reads). Other examples included expression of genes encoding homologs to an hypothetical protein A7U43_25800 of *Mycobacterium sp. YC-RL4* (147 reads), an arylsulfatase regulatory protein of *Sphingobium ummariense RL-3* (45 reads), a NADPH dehydrogenase NamA of *Chitinophagaceae bacterium PMP191F* (37 reads), and a transposase of *Salmonella enterica* subsp. *enterica* serovar Heidelberg *str. SL476* (31 reads) (Table 2).

**Table 2:** Examples of highly expressed genes best matching with bacterial genes.

Gene id*	Best hit	Function	Organism	Reads count
g6416	WP_014678436.1	Tandem-95 repeat protein	*Solitalea canadensis*	349
g2762	ANE82214.1	Hypothetical protein A7U43_25800	*Mycobacterium* sp. YC-RL4	147
g4799	EQB29884.1	Arylsulfatase regulatory protein	*Sphingobium ummariense* RL-3	45
g11285	WP_054281538.1	NADPH dehydrogenase NamA	*Chitinophagaceae* bacterium PMP191F	37
g4808	ACF68028.1	Transposase	*Salmonella enterica* subsp. *enterica* serovar Heidelberg str. SL476	31

*In *V. vermiformis* .

**Figure 3:**
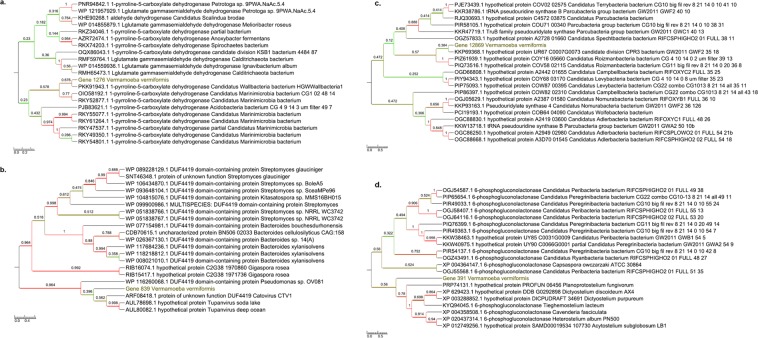
Phylogenetic reconstructions for four examples of putative lateral sequence transfers implicating *V. vermiformis* and bacteria. Lateral sequence transfer was inferred from the comparison of *V. vermiformis* predicted sequences with their best BLAST hits. (**a**,**b**) Trees based on two proteins with sequence similarity with a non-CPR bacterial homolog. (**c**,**d**) Trees based on two proteins with sequence similarity with a CPR homolog. In dark yellow: *V. vermiformis genes*. Colors of branches are related to bootstrap values.

Furthermore, 30 genes best matching with members of the Candidate Phyla Radiation (CPR) were detected in the genome of *V. vermiformis* CDC-19, which is equal to 1.3% of the total set of homologs to bacterial genes. These genes were primarily related to the *Parcubacteria* group (22 genes), then to the *Microgenomates* group (5 genes), and to CPR2, *Peregrinibacteria* and *Doudnabacteria* (with 1 gene related to each CPR phylum) (Fig. 2; Supplementary Table S3). A majority of the genes best matching with CPR corresponded to hypothetical proteins (23 genes (76.6%)), whereas some were found to be involved in carbohydrate transport and metabolism (1 gene; a 6-phosphogluconolactonase), nucleotide transport and metabolism (1 gene), translation, ribosomal structure and biogenesis (1 gene), cell wall, membrane, and envelope biogenesis (1 gene), and in undetermined functions (3 genes) (Supplementary Table S3). Phylogenetic reconstructions confirmed that these genes underwent sequence transfers between *V. vermiformis* and bacteria (Fig. 3; Supplementary Fig. S4), and one of them was found to be expressed (Fig. 3a). Moreover, there was at least on example of a DUF4419 domain-containing protein that might have been thereafter transferred to Catovirus CTV1 and Tupanvirus, two giant viruses (Fig. 3b). The same observations were obtained for genes showing sequence similarity with a CPR homolog (Fig. 3c,d).

### Sequence exchanges between *V. vermiformis* CDC-19 and viruses

**Figure 4:**
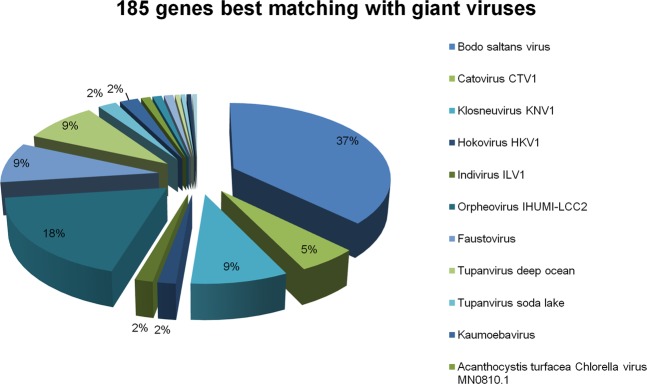
Taxonomical origins of predicted genes with a giant virus as best hit.

**Figure 5:**
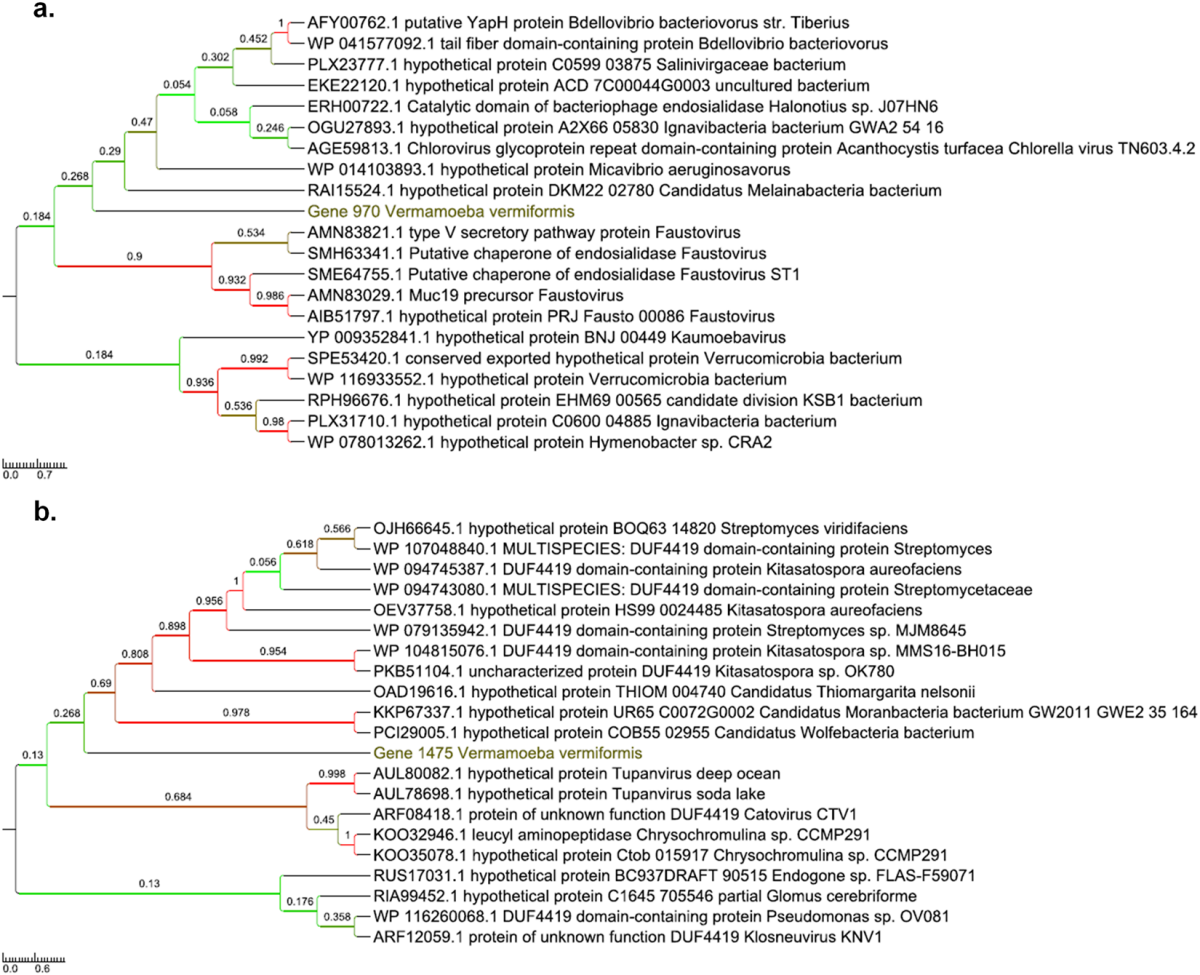
Phylogenetic reconstructions for two examples (**a**,**b**) of putative lateral sequence transfers implicating *V. vermiformis* and giant viruses. Lateral sequence transfer was inferred from the comparison of *V. vermiformis* predicted sequences with their best BLAST hits. In dark yellow: *V. vermiformis genes*. Colors of branches are related to bootstrap values.

**Figure 6:**
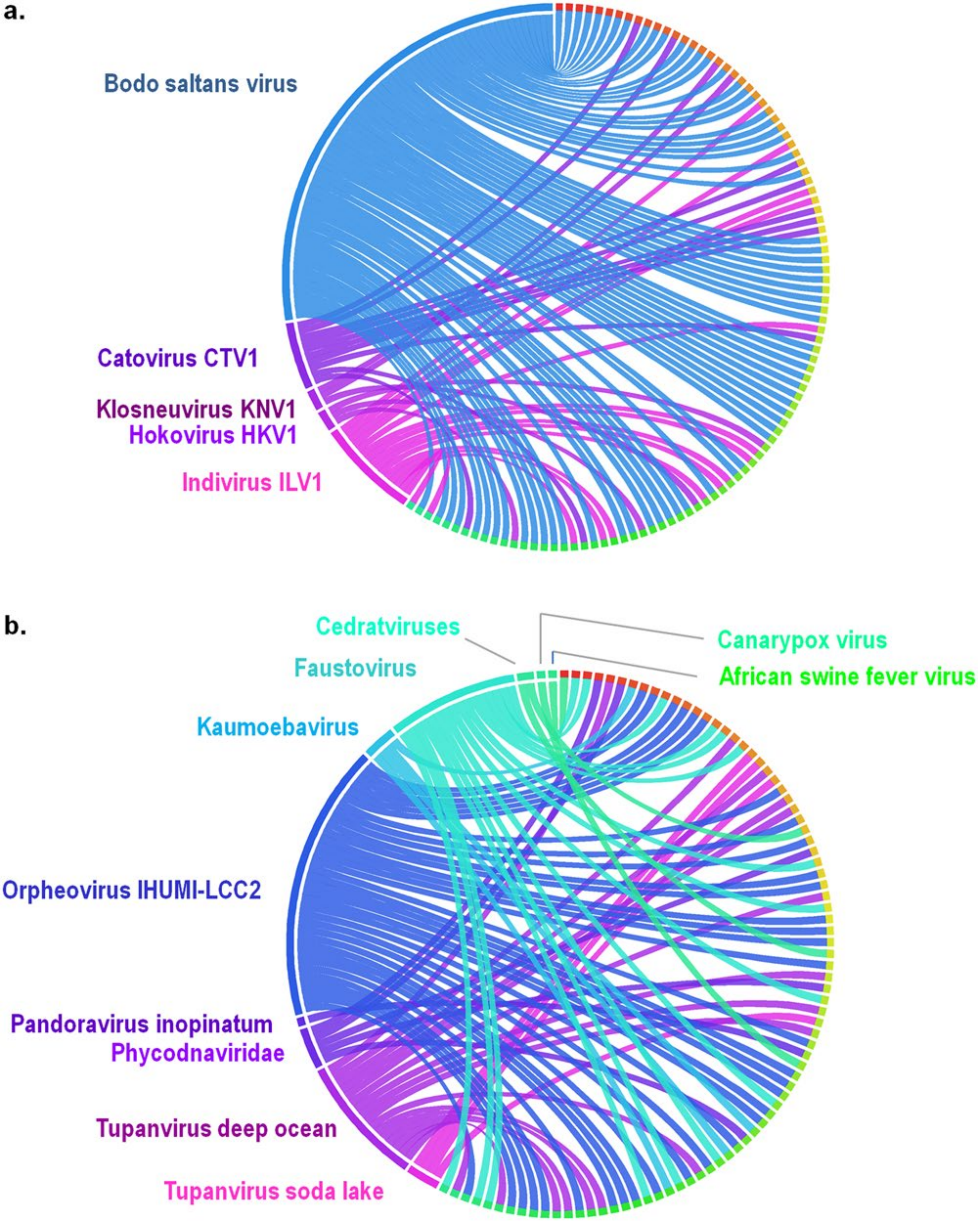
Rhizomes representation of the proteins best matching with giant viruses. Taxonomical distribution of *V. vermiformis* CDC-19 predicted proteins for which best BLAST hits were members of family *Mimiviridae* (**a**) and other giant viruses (**b**).

Of the 188 genes detected in the genome of *V. vermiformis* CDC-19 that best matched with viruses, 185 of these best matched with giant viruses. The three other genes best matched with Ralstonia phage phiRSL1 (2 genes) and Synechococcus phage S-SKS1(1 gene), and encode hypothetical proteins. Genes best matching with giant viral genes were mostly related to Megavirales members, such as those best matching with Bodo saltans virus, a member of family *Mimiviridae*, subfamily *Klosneuvirinae* (69 genes) (Fig. 4). Other best matches were genes from assembled genomes of putative members of the *Klosneuvirinae*: their best hits were with genomes of Klosneuvirus KNV1 (16 genes), Catovirus CTV1 (10 genes), Hokovirus HKV1, and Indivirus ILV1 (3 genes each). Other homologs were from two mimivirus isolates, Tupanvirus deep ocean (16 genes) and Tupanvirus soda lake (4 genes), which replicate in *A. castellanii* and *V. vermiformis*. Viral sequences from other Megavirales groups than *Mimiviridae* were also identifed as best hits, such as genes from Orpheovirus IHUMI-LCC2 (34 genes), faustoviruses (16 genes), Kaumoebavirus (4 genes), cedratviruses (2 genes), and Pandoravirus inopinatum (1 gene). In addition, 5 *V. vermiformis* genes best matched with phycodnavirus genes, 4 of them belonging to Acanthocystis turfacea Chlorella viruses MN0810.1 and WI0606, and one gene best matched with Phaeocystis globosa virus. A homolog was also detected in Canarypox virus and African swine fever virus (Supplementary Table S4). Phylogenies strengthened suspicions of lateral sequence transfer for two genes best matching with giant viruses (Fig. 5). At least one gene of *V. vermiformis* best matched with viral sequences as well as with CPR and other bacteria (Fig. 5b). A total of 70 of the 185 genes best matching with giant viruses encode ankyrin repeat domain-containing proteins. The majority of these genes (68) were homologs to Bodo saltans virus genes, and the two other genes were homologous to Klosneuvirus KNV1 and Canarypox virus genes. Eighteen genes encode proteins with a DUF4114 domain and were related to Orpheovirus IHUMI-LCC2 (16 genes) and Catovirus CTV1 (2 genes). Gene expression was detected for nine genes best matching with giant viral sequences. Eight genes were related to Orpheovirus IHUMI-LCC2 and notably encode a DUF4114 protein and an E-class cytochrome P450-like protein. Among remaining best matches was a gene of Kaumoebavirus predicted to encode a peroxinectin, which was the first gene encoding cell adhesion ligand and peroxidase molecule cloned from invertebrate blood^40^ (Table 3). Finally, the rhizomes of *V. vermiformis* CDC-19 genes best matching with *Klosneuvirinae* representative sequences demonstrated that sequence exchanges between *V. vermiformis* CDC-19 and each member of this subfamily were widely distributed on different scaffolds of the amoebal draft genome sequence (Fig. 6a). Similar observations were found for genes best matching with sequences from other Megavirales members, such as Orpheovirus IHUMI-LCC2, faustoviruses, and tupanviruses (Fig. 6b).

**Table 3:** Nine examples of expressed genes best matching with viral sequences, including Orpheovirus IHUMI-LCC2 and Kaumoebavirus.

Gene id*	Best hit	Function	Organism
g4093	YP_009449258.1	Domain of unknown function (DUF4114)	Orpheovirus IHUMI-LCC2
g4206	YP_009449258.1	Domain of unknown function (DUF4114)	Orpheovirus IHUMI-LCC2
g10030	YP_009449258.1	Domain of unknown function (DUF4114)	Orpheovirus IHUMI-LCC2
g12378	YP_009448979.1	Cytochrome P450-like protein E-class	Orpheovirus IHUMI-LCC2
g13490	YP_009449258.1	Domain of unknown function (DUF4114)	Orpheovirus IHUMI-LCC2
g13679	YP_009449258.1	Domain of unknown function (DUF4114)	Orpheovirus IHUMI-LCC2
g14004	YP_009448979.1	Cytochrome P450-like protein E-class	Orpheovirus IHUMI-LCC2
g15726	YP_009352567.1	Peroxinectin	Kaumoebavirus
g18288	YP_009449258.1	Domain of unknown function (DUF4114)	Orpheovirus IHUMI-LCC2

*In *V. vermiformis*.

## Discussion

We herein describe for the first time the genome sequencing, composition and characteristics for a strain of the amoeba *Vermamoeba vermiformis* CDC-19. This draft genome sequence is larger than those of other amoebae such as *Naegleria gruberi* and *Acanthamoeba castellanii Neff*, which are estimated to be equal to 41 and 42 Mbp, respectively^29,41^. It is comprised by 14,852 scaffolds, fewer than previously described for the *Acanthamoeba* spp. draft genome sequence. One third of predicted genes in this *V. vermiformis* strain were ORFans, which leaves questions surrounding the repertoire of the genes and their roles. In addition, a large proportion of the non-ORFan genes was found to encode unknown functions based on comparative analyses with COGs. On average, *V. vermiformis* genes were found to contain 3.5 introns whereas *A. castellanii* Neff genes harbor 6.2 introns^29^. The difference between these amoebae may reflect extensive intron losses or gains, and supports the importance of introns in evolution^42^. The prevalence of introns in genes involved in sequence tranfers with bacteria and giant viruses implies the proposed mechanisms of intron gain subsequently to lateral sequence transfer^43^.

More than half of *V. vermiformis* CDC-19 predicted genes have eukaryotic sequences as closest relatives. Approximately 10% of genes have bacterial sequences as best hits, a proportion similar to that described for *A. castellanii* Neff^29^. However, the proportion of genes best matching with bacteria was greater than for other described amoebae, as for *A. polyphaga* (3%)^31^ or *Naegleria gruberi* (1%)^41^. The presence in the *V. vermiformis* CDC-19 genome of sequences that were predicted to have resulted from exchanges with amoeba-resisting microorganisms, particularly bacteria, was confirmed by Sanger sequencing in all cases when tested for a small set of genes. In addition, transcriptomics showed expression of several genes best matching with bacterial sequences, the highest level of gene expression being observed for a gene encoding a tandem-95 repeat protein. Classically, tandem repeats act as a support for protein-protein interactions, but it has been hypothetized that the gain or loss rates of such sequences might generate genetic diversity and evolutionary adaptation to a pathogen^44^. The other transcribed genes best matching with bacteria were mainly related to either undetermined functions, or to replication, recombination and repair, and energy production and conversion pathways. As in the study of Clarke *et al*. on the draft genome sequence of *A. castellanii* Neff, sequences best matching with genes from members of phyla *Proteobacteria, Bacteroidetes* and *Cyanobacteria* were those the most represented in the genome of *V. vermiformis* CDC-19^29^. However, the proportion of genes best matching with *Bacteroidetes* and *Cyanobacteria* members was slightly greater (2% and 3%, respectively) for *V. vermiformis* CDC-19, when compared to *A. castellanii* Neff.

We reported here the first identification in an amoebal genome of sequences best matching with CPR. CPR were recently described as small bacteria that may represent >15% of all bacterial diversity and dozens of phyla^45^. It is likely that they have been previously overlooked because of their small size, and they have small genomes and an apparent symbiotic lifestyle with bacteria^46,47^. They have been detected in a wide range of natural systems, including groundwaters and sediments. Sequences from CPR were only recently available in the NCBI database, which prevented their earlier detection. CPR homologs encompassed 1.3% of the gene products best matching with bacteria in the genome of *V. vermiformis*. These data highlight a yet unexplored gene trafficking between CPR and *V. vermiformis*.

A set of 188 genes in *V. vermiformis* CDC-19 was related to sequences from viruses, essentially giant viruses. Their number was smaller, albeit similar, compared to those reported for *A. castellanii Neff* (261)^30^ or *A. polyphaga* draft genomes (262)^31^. These genes were detected in a large set of 179 scaffolds comprising the draft genome sequence of *V. vermiformis* CDC-19, suggesting that they are widely distributed along the genome of this amoeba. We demonstrated that the genomes of klosneuviruses, particularly that of Bodo saltans virus, harbored the largest set of such virus-related sequences. This suggests a considerable gene trafficking between this amoeba and *Klosneuvirinae* members. Among this group, only the Bodo saltans virus was isolated (only the genomes assembled from metagenomic data being available for the other described members) and this was on the kinetoplastid *Bodo saltans*, a microzooplankton^48^. Other recently described mimiviruses named tupanviruses can grow on both *Acanthamoeba* spp. and *V. vermiformis*^37^. However, most commonly, the permissivity of known eukaryotic hosts to giant viruses differs considerably according to the host strain or to the viral family or lineage, as previously described for mimiviruses, pandoraviruses, and Bodo saltans virus^32,48^. The analysis of giant virus homologs in the *V. vermiformis* genome showed here that the most represented sequences were those of giant viruses that grew in *V. vermiformis*, including faustovirus isolates and Orpheovirus IHUMI-LCC2, whereas a small proportion included genes from giant viruses isolated from *Acanthamoeba* spp.. Ankyrin repeats, which are associated with protein-protein interactions, were highly represented among *V. vermiformis* genes best matching with giant viruses^49,50^ in addition to DUF4114 domains which are conserved domains that help to adapt to nutrient-depleted conditions by down-regulating protein biosynthesis^51^. Overall, the phylogenies of genes predicted to have arisen through lateral sequence transfer illustrate the complexity of sequence exchanges between amoebae, bacteria (including CPR), and giant viruses. This result is in line with the recent analysis of *Acanthamoeba* genomes, suggesting that the sequence flow was not a one way mechanism, and a possible result of their sympatric lifestyle^30,31^.

Overall, these first *V. vermiformis* genome-wide genetic data allow for a better understanding of this amoeba and its interactions with microorganisms. They provide insight on an extensive gene trafficking with distinct amoeba-resisting microorganisms, including bacteria and giant viruses. They also suggest as expected that the presence of genes from these microorganisms in cellular genomes are hints that these cells are among their possible hosts. Moreover, the comparison of different amoebal genomes and gene repertoires is an important task that might help us understand the different levels of their susceptibility to giant viruses, and select efficient cellular supports for their isolation.

## Materials and Methods

### Vermamoeba vermiformis strain CDC-19 culture

*Vermamoeba vermiformis* strain CDC-19 was isolated from cooling tower water in a hospital during a legionellosis investigation^20^. This strain was obtained from the American Type Culture Collection database (ATCC). *V. vermiformis* CDC-19 (ATCC 50237) was grown at 32 °C in 175 cm² culture flasks (Thermo Fisher Scientific, Illkirch, France) containing 75 mL of PYG medium^52^. When amoebas formed a monolayer, they were detached by tapping the culture flasks then harvested by centrifugation at 1,000 g for 10 min followed by three steps of washing using Page’s modified Neff’s Amoeba Saline medium (2 mM NaCl, 16 μM MgSO_4_, 27.2 μM CaCl_2_, 1 mM Na_2_HPO_4_, 1 mM KH_2_PO_4_). Strain CDC-19 quantification was performed using a KOVA slide cell counting chamber.

### Genomic DNA extraction and sequencing of the amoeba *V. vermiformis* CDC-19

The DNA of *V. vermiformis* CDC-19 was extracted using the EZ1 DNA Tissue Kit (Cat No: 953034, Qiagen, Hilden, Germany), then purified using the Agencourt AMPure XP beads (1.8x ratio, Beckman Coulter Inc, Fullerton, CA, United States). Genomic DNA was quantified by a Qubit assay with the high sensitivity kit (Life technologies, Carlsbad, CA, USA); the concentration was equal to 2.3 ng/µl. A dilution was performed to provide 1 ng of DNA as input to prepare the paired end library. The «tagmentation» step fragmented and tagged the DNA and limited cycle PCR amplification (12 cycles) completed the tag adapters and introduced dual-index barcodes, in order to allow mixing with other genomic projects. After purification on AMPure XP beads (Beckman Coulter Inc), the libraries were normalized on specific beads according to the Nextera XT DNA sample prep kit protocol (Illumina, San Diego, CA, USA). Normalized libraries were pooled into a single library for sequencing on the MiSeq instrument (Illumina). Automated cluster generation and paired-end sequencing with dual index reads were performed in a 39-hour run with 2 × 250 bp. To improve the assembly, the Oxford Nanopore technology (Oxford Nanopore Technologies Ltd., United Kingdom) was used by 1D genomic DNA sequencing on the MinION device using the SQK-LSK108 kit. The library was constructed from 1.5 µg of genomic DNA without fragmentation and end repair. Adapters were ligated to both ends of genomic DNA. After purification on AMPure XP beads (Beckman Coulter Inc), the library was quantified by a Qubit assay with the high sensitivity kit (Life technologies), and loaded on the flow cell via the SpotON port. Finally, 498 active pores were detected for the sequencing and the workflow WIMP was chosen for sequence analysis. Adapter trimming, quality filtering and error correction of all sequencing raw data analyzed here were performed using the Trimmomatic program (version 0.36)^53^.

### Total RNA preparation and sequencing

The RNA of *V. vermiformis* CDC-19 was extracted using the RNeasy mini kit (Cat No: 74104, Qiagen). RNaseOUT (Thermo Fisher Scientific, San Jose, CA, USA) was added to the 50 μL volume of eluted RNA, thus preventing RNA degradation. To ensure of the absence of DNA contamination, two cycles of DNase treatment with 30 min of incubation at 37 °C were performed using TURBO DNase (Invitrogen, Carlsbad, CA, USA). Total RNA was purified using the RNeasy MinElute Cleanup Kit (Cat No: 74204, Qiagen) according to the manufacturer’s instructions. cDNA amplicons were obtained using the SuperScript VILO Synthesis Kit (Invitrogen) with random primers. The amplicons were purified using the Agencourt AMPure XP beads (Beckman Coulter, Inc.), then sequenced on the MiSeq instrument using the Nextera XT DNA sample prep kit (Illumina), with a paired-end strategy and a read length of 125 bp. The cDNA was visualized and quantified on a LabChip Bio-analyzer (Agilent Technologies). Fragmentation, tagging and barcoding were performed over 12 PCR amplification cycles. The library was purified on Agencourt AMPure XP beads (Beckman Coulter Inc.), normalized on specific beads, and pooled for sequencing.

### Assembly of the *V. vermiformis* CDC-19 genomic sequences

We assembled the genome of *V. vermiformis* CDC-19, whose ploidy was estimated to be 4 N^5,54^ using the A5-miseq pipeline, which included supplementary steps of adapter trimming and quality filtering^55^. Although the A5 software was classically used for bacterial and haploid organisms, it was also used for polyploid eukaryotic organisms (including *Verticillium tricorpus* and *Verticillium dahliae)* and allowed obtaining assemblies of high quality^56^. The quality assessment of the genome asssembly was performed using the QUAST software^57^. MinION fastq reads were assembled separately using the SPAdes program^58^. Thereafter, mapping of both MiSeq and MinION contigs was performed using the CLC Genomics Workbench software (version 7.5) (https://www.qiagenbioinformatics.com/products/clc-genomics-workbench/), followed by manual treatment to detect consensus sequences and gaps filling on the resulting genomic sequences of *V. vermiformis* CDC-19 using the GapFiller program^59^. A phylogenetic analysis based on the 18S rRNA gene was performed. For this task, we detected the 18S rRNA gene of *V. vermiformis* CDC-19 by comparison through BLASTn between the amoebal genome assembly and the published 18 s rRNA sequence of another *V. vermiformis* strain (KY476315.1), and also searched for similar sequences in the NCBI GenBank nucleotide sequence collection (nt). We then carried out multiple sequence alignments by using the MEGA version 7 software^60^. Finally, we performed a phylogenetic reconstruction of these nucleotide sequences using MEGA7^60^ and the maximum likelihood (ML) algorithm, with 1,000 replicates for bootstrap determination.

### Prediction, expression assessment, taxonomical distribution, and functional annotation of the *V. vermiformis* CDC-19 putative genes

Prediction of putative genes was implemented using the BRAKER1 program^61^ based on the genomic sequences and the RNA-seq raw data of *V. vermiformis* CDC-19. An additional mapping of the RNA-seq reads on the assembled genome was performed by using the HISAT2 software^62^, with default parameters. The reads aligned on the amoebal genome sequence were analyzed using HTSeq-count software^63^, with union mode. Predicted genes were estimated as transcribed if covered by at least 5 reads. In addition, we searched for homologous sequences of predicted open reading frames (ORFs) in the NCBI GenBank protein sequence database (nr) using the BLASTp program, with an e-value threshold of 0.001 and default parameters (word size equal to 3, gap costs equal to 11 for the opengap parameter and 1 for the gap extend parameter)^64^. To ensure the absence of suspected contaminant reads in the genome of *V. vermiformis* CDC-19, scaffolds harboring a totality of their genes best matching with the same bacteria were excluded. Sequences homologies were also identified using the eggNOG-mapper through searches using DIAMOND in the NCBI COG (Clusters of Orthologous Groups of proteins) database^65–67^. Finally, taxonomical assignments were deduced using the MEGAN6 program^68^.

### Detection of sequence exchanges between *V. vermiformis* CDC-19 and other microorganisms

The representations as a ‘rhizome’ of the repertoire of genes predicted for *V. vermiformis* CDC-19 that best matched with sequences from giant viruses were built using the Circos tool (http://circos.ca/). Rhizomes aim to display genome mosaicism. Here, rhizomes of genes were built by BLASTp searches with complete sequences of these genes. The number and taxonomical assignments of *V. vermiformis* CDC-19 genes best matching with bacterial sequences were determined using the MEGAN6 program^68^. We randomly extracted the nucleotide sequences of 10 genes best matching with bacteria that were found to be co-localized with other genes of *V. vermiformis* CDC-19 at different positions in its genome. PCR primer systems were designed in order to target the region that straddles a gene best matching with bacteria and a gene of the amoeba, using the Primer3Plus program^69^. *V. vermiformis* CDC-19 DNA was amplified during 35 PCR cycles with the ten primer systems separately and the AmpliTaq Gold 360 Master mix (Applied Biosystems, Foster City, CA, USA; ref. 4398881). PCR products were purified using the Nucleofast 96 PCR clean-up kit (Macherey Nagel, Düren, Germany; ref. 743100). Purified products were sequenced using the BigDye Terminator V1.1 Sequencing Kit (Applied Biosystems; ref. 4336776), with a Sanger sequencing method on an ABI-3130 XL genetic analyser (Applied Biosystems). Finally, phylogenetic analyses were performed to strengthen the evidence of lateral sequence transfer for four genes whose presence was confirmed by PCR and Sanger sequencing and that have a bacterial homolog, two genes that had as top BLASTp hits a CPR homolog and two genes best matching with giant viral homologs. After amino acid sequence alignment with corresponding best hits using the MUSCLE program^70^, phylogenetic reconstructions were performed using the MEGA6 program, with a Maximum Likelihood method (http://www.megasoftware.net/).

## Supplementary information

Supplementary Dataset 1.

Supplementary Dataset 2.

Supplementary Dataset 3.

Supplementary Dataset 4.

Supplementary Dataset 5.
