# Supplementary material for: Vermamoeba vermiformis CDC-19 draft genome sequence reveals considerable gene trafficking including with candidate phyla radiation and giant viruses
Source: Sci Rep. 2020 Apr 3;10:5928. doi: 10.1038/s41598-020-62836-9 (PMC7125106; doi:10.1038/s41598-020-62836-9)
Supplement: Supplementary file 1 — Supplementary Dataset 1. [file 41598_2020_62836_MOESM1_ESM.docx]

**TITLE PAGE FOR THE SUPPLEMENTARY INFORMATION**

**Full-length title: *Vermamoeba vermiformis* CDC-19 draft genome sequence reveals considerable gene trafficking including with candidate phyla radiation and giant viruses**

Short title (for the running head): *Vermamoeba vermiformis* CDC-19 draft genome sequence

**Authors list: Nisrine CHELKHA^1,2^, Issam HASNI^1,2,3^, Amina CHERIF LOUAZANI^1,2^, Anthony LEVASSEUR^1,2^, Bernard LA SCOLA^1,2^*, Philippe COLSON^1,2^***

**Affiliations:** ^1^ Aix-Marseille Université, Institut de Recherche pour le Développement (IRD), Assistance Publique - Hôpitaux de Marseille (AP-HM); Microbes, Evolution, Phylogeny and Infection (MEPHI); Institut Hospitalo-Universitaire (IHU) - Méditerranée Infection, 27 boulevard Jean Moulin, 13005 Marseille, France; ^2^ IHU Méditerranée Infection, 19-21 boulevard Jean Moulin, 13005 Marseille, France; ^3^ Amoéba, 38 avenue des Frères Montgolfier, 69680 Chassieu, France.

*** Corresponding authors:** Philippe Colson and Bernard La Scola, IHU - Méditerranée Infection, AP-HM, 19-21 boulevard Jean Moulin, 13005, Marseille, France. E-mail addresses: philippe.colson@univ-amu.fr; bernard.la-scola@univ-amu.fr

**Supplementary figures**

**Supplementary Figure S1. Phylogenetic tree based on 18S rRNA of *Vermamoeba vermiformis* CDC-19.** GenBank Accession numbers are indicated in parentheses. The sequences were aligned by MEGA7 and tree was performed using Jukes-Cantor model on MEGA 7.0.25 software. Numbers at the nodes are percentages of bootstrap values obtained by repeating the analysis 1,000 times to generate a consensus tree; only values ≥ 0,7 were displayed. The scale bar indicates a 10% nucleotide sequence divergence.

**
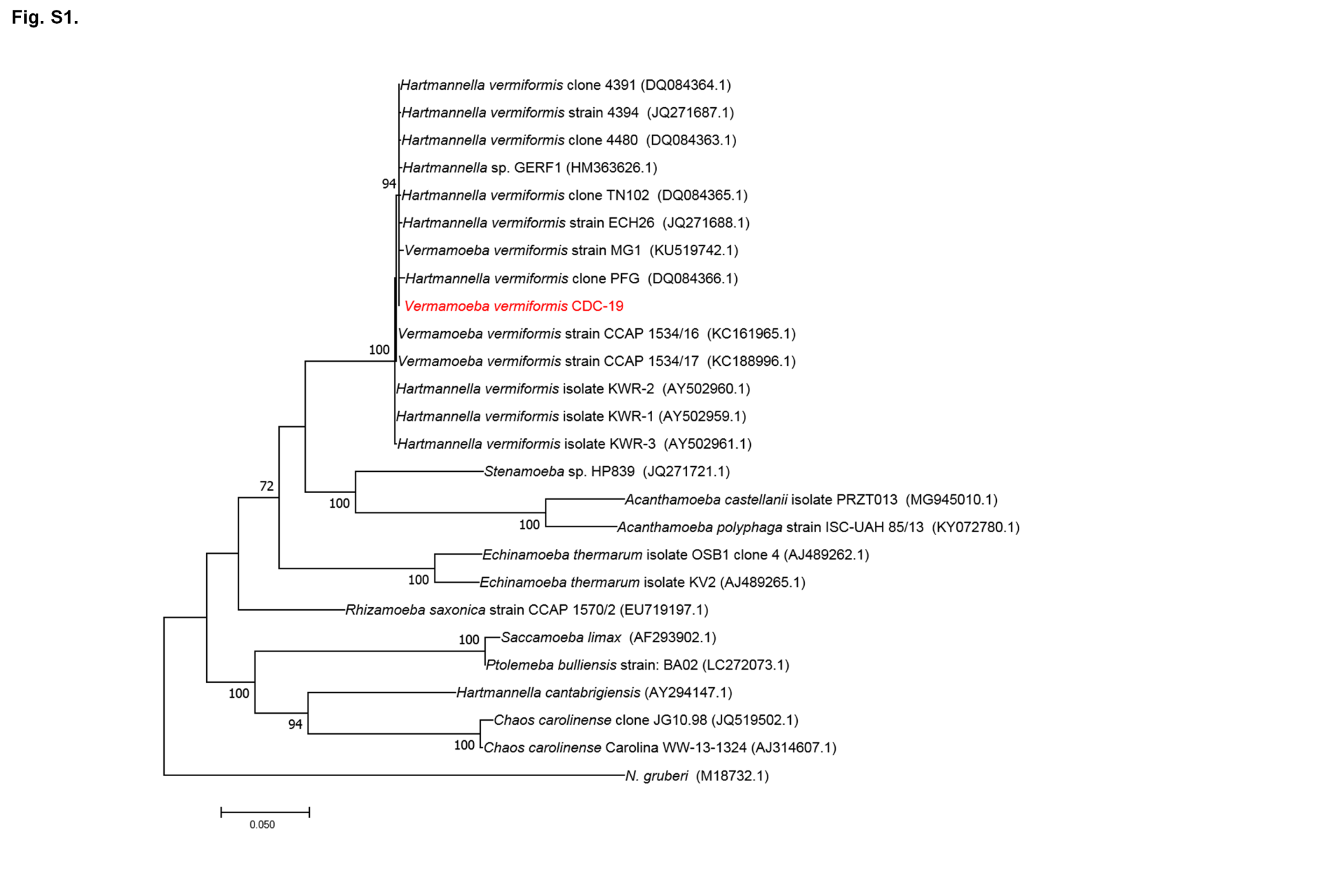
**

**Supplementary Figure S2.** **Distribution of the predicted genes from *V. vermiformis* CDC-19 in categories of clusters of orthologous groups of proteins (COGs).**

**
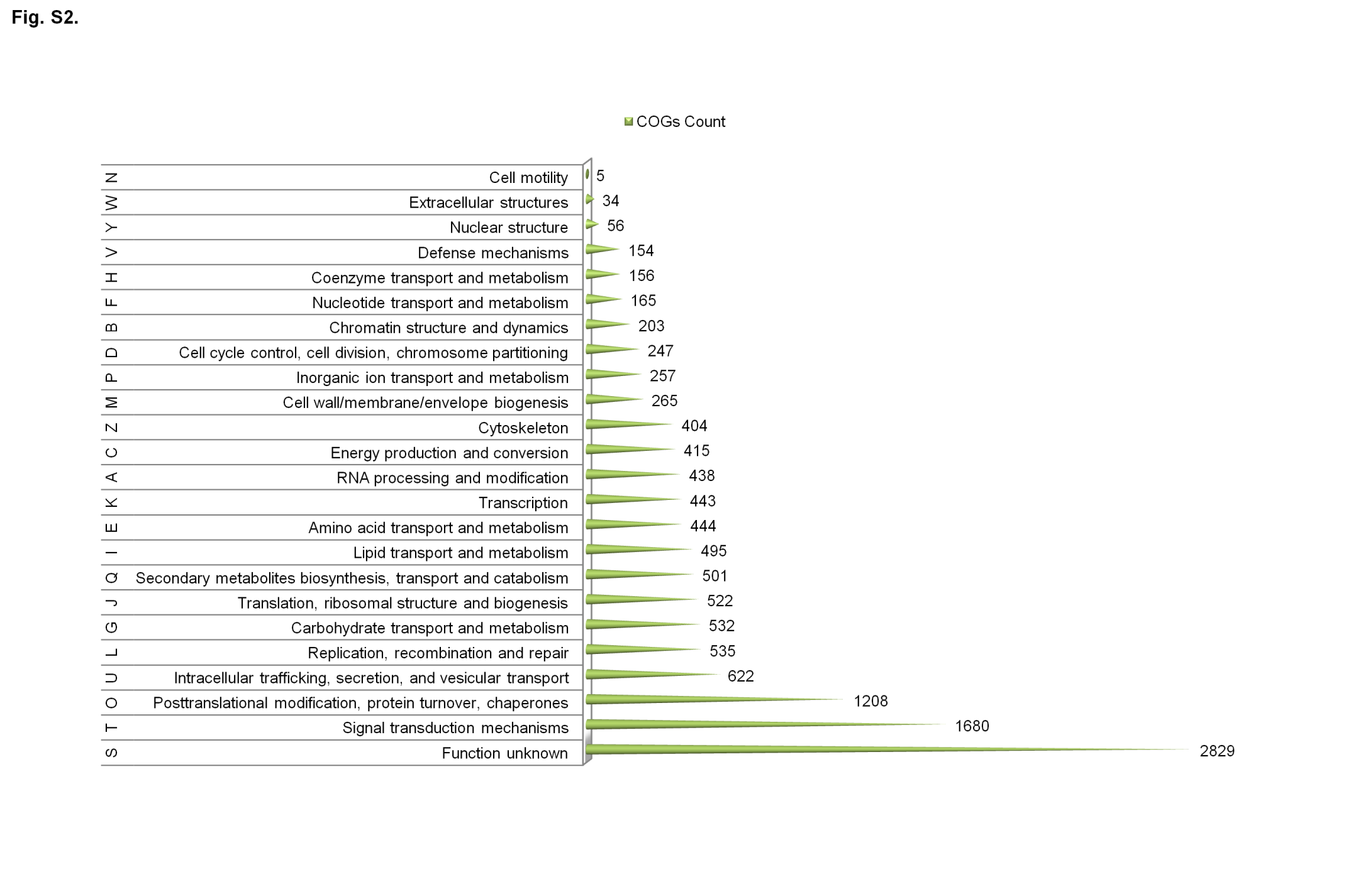
**

**Supplementary Figure S3.** **Sequencing results for ten genes best matching with bacteria in the genome of *V. vermiformis*.**

a: alignment of the predicted sequence and Sanger sequencing result for the region that straddles in the of the amoebal genome the gene 373 and the gene 374 that best matches with a bacterial gene. b-j: similar alignments for ten other examples of *V. vermiformis* genes best matching with bacterial genes.

**
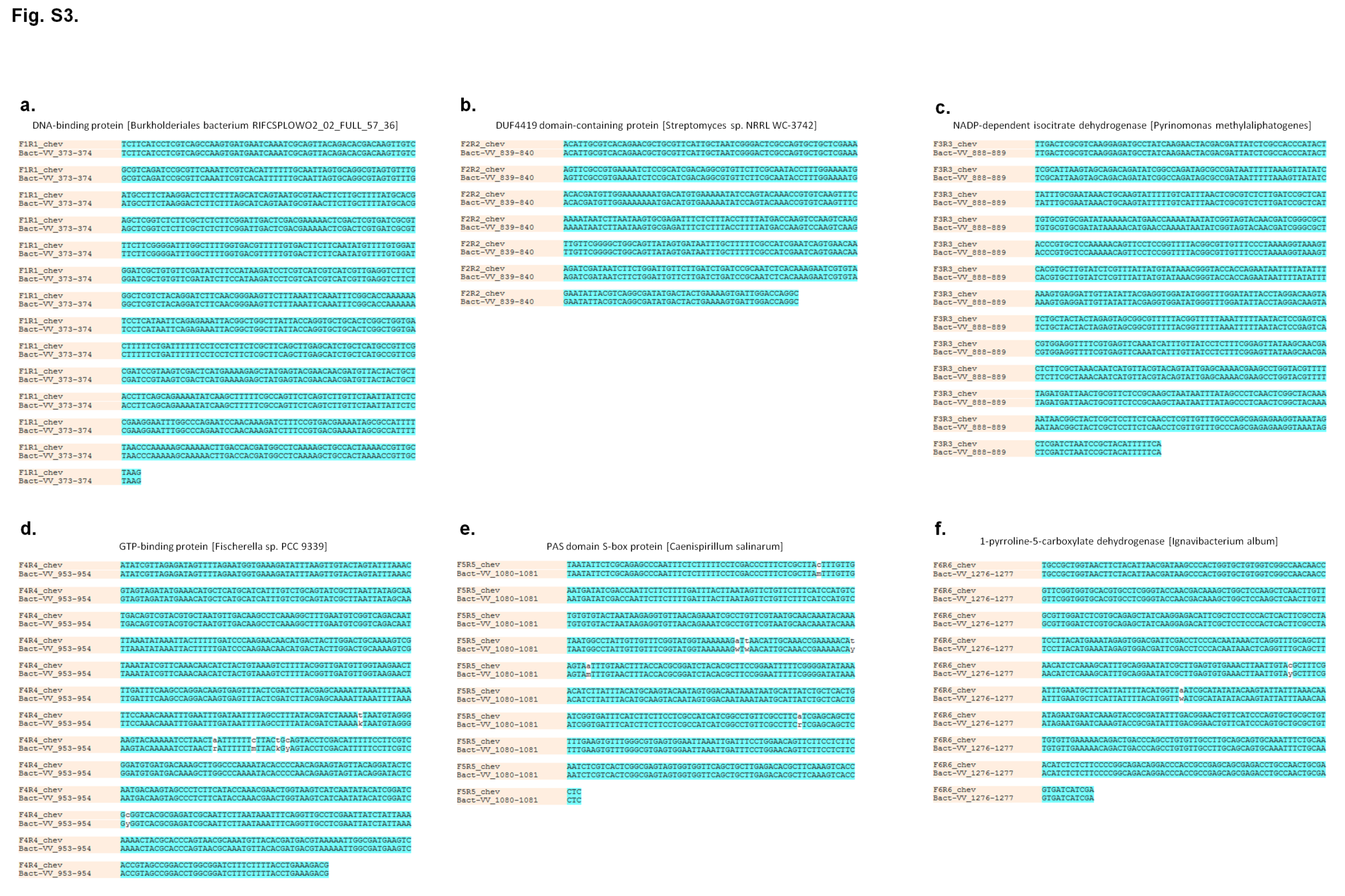
**

**
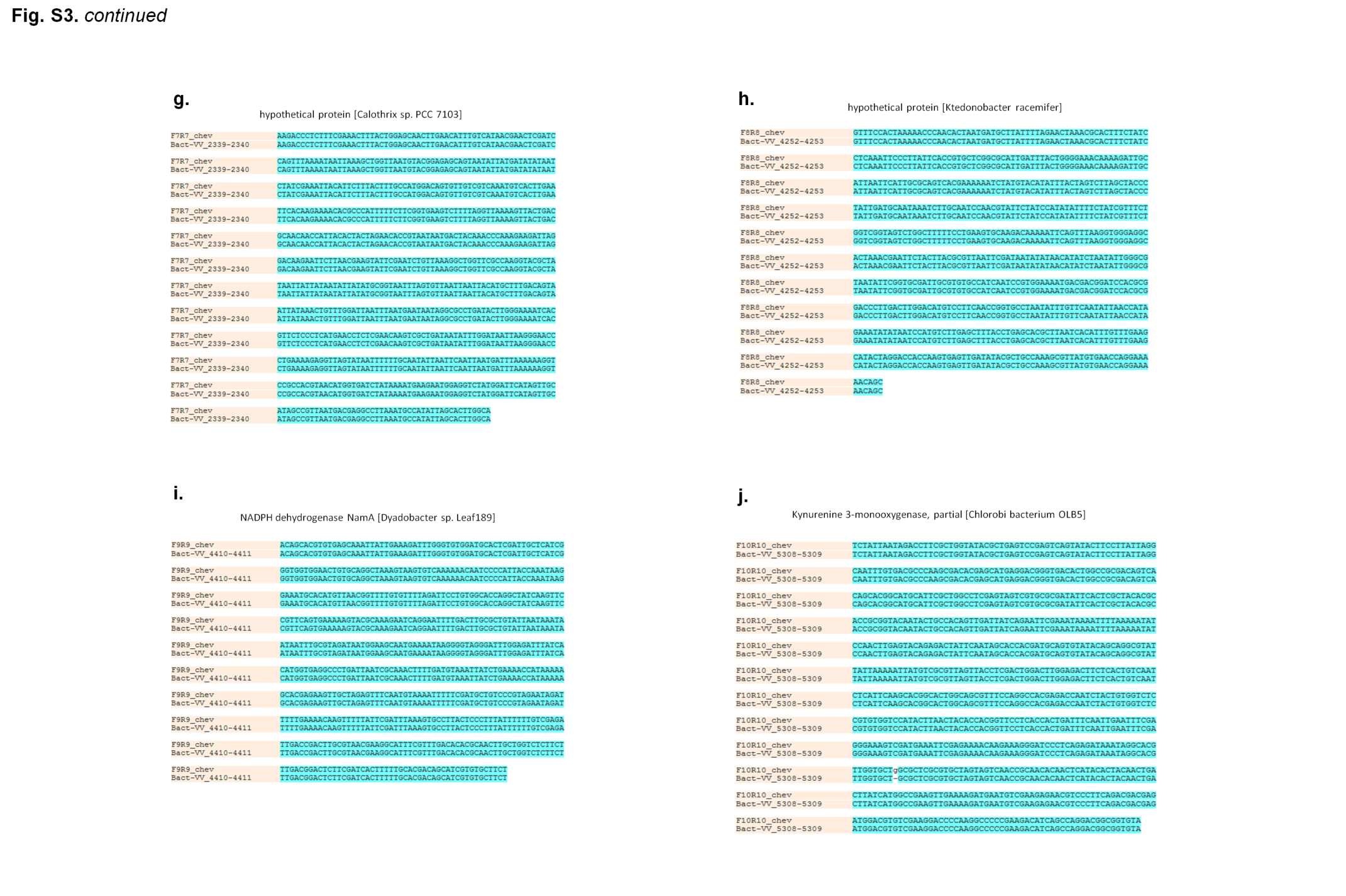
**

**Supplementary Figure S4.** **Phylogenetic reconstructions for two additional examples (a, b) of putative lateral sequence transfers implicating *V. vermiformis* and bacteria.**

Lateral sequence transfer was inferred from the comparison of *V. vermiformis* predicted sequences with their best BLAST hits*.* In dark yellow: *V. vermiformis genes.* Colors of branches are related to bootstrap values.

**
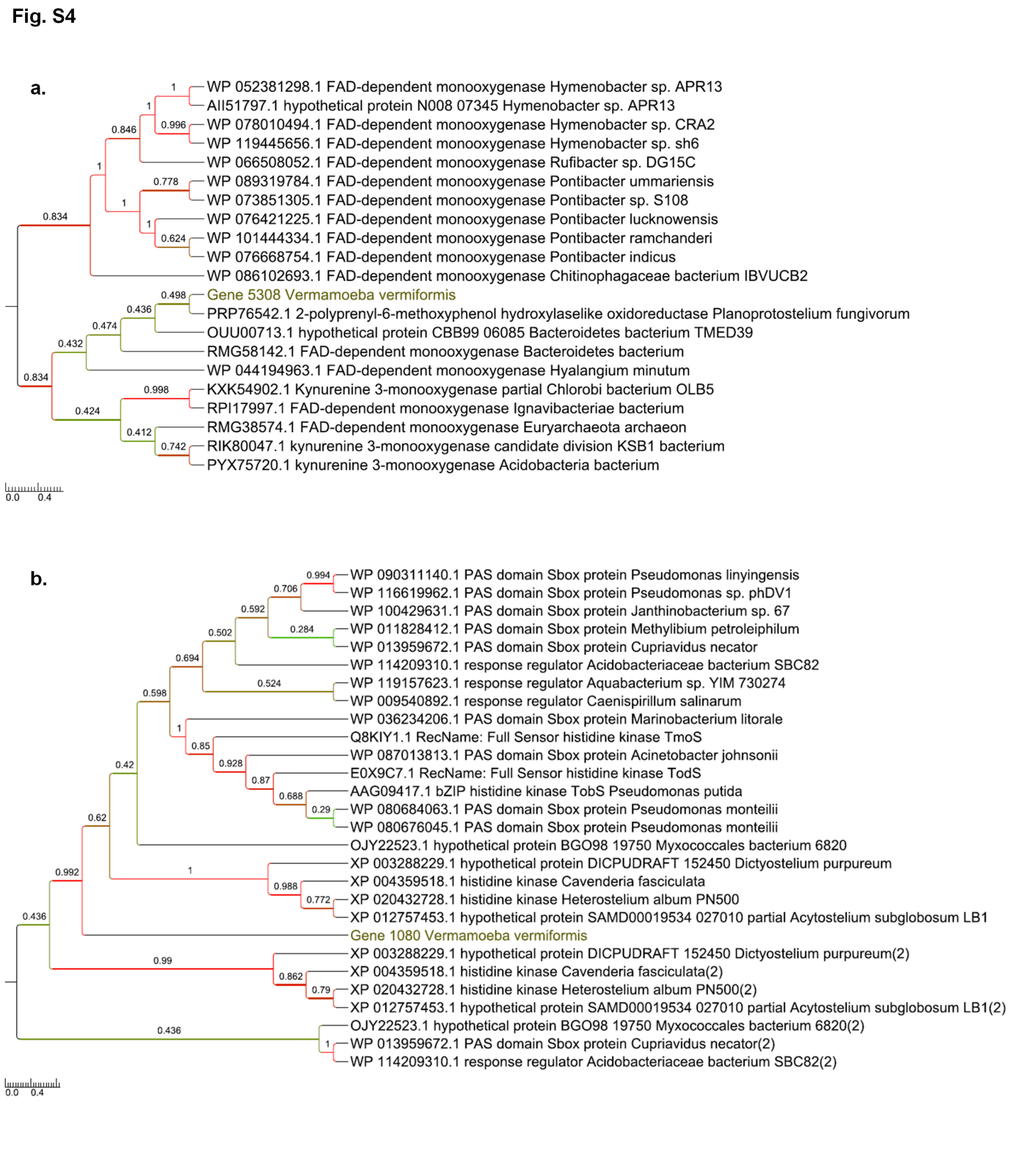
**

**Supplementary tables**

**Supplementary Table S1.** Assignment of V. vermiformis CDC-19 predicted genes best matching with bacteria to categories of clusters of orthologous groups of proteins (COGs).

**Supplementary Table S2. PCR primers used to target in the genome of *V. vermiformis* ten genes best matching with bacterial genes (see also Supplementary Figure S2).**

**Supplementary Table S3. *V. vermiformis* genes best matching with genes from bacteria classified as Candidate Phyla Radiation (CPR).**

**Supplementary Table S4. *V. vermiformis* genes best matching with genes from giant viruses.**
